# Supplementary material for: Pyruvate Dehydrogenase Kinase 1 inhibition mediated oxidative phosphorylation enhancement in cartilage promotes osteoarthritis progression
Source: BMC Musculoskelet Disord. 2023 Jul 20;24:597. doi: 10.1186/s12891-023-06585-6 (PMC10357736; doi:10.1186/s12891-023-06585-6)
Supplement: Supplementary file 1 — Additional file 1. [file 12891_2023_6585_MOESM1_ESM.zip › KEGG.Gsea.1653622432464/KEGG_GLYCOSAMINOGLYCAN_DEGRADATION.html]

Details for gene set KEGG\_GLYCOSAMINOGLYCAN\_DEGRADATION[GSEA]

|  || Dataset | 55457\_collapsed\_to\_symbols.cls.cls #OA\_versus\_normal.cls.cls #OA\_versus\_normal\_repos |
| Phenotype | cls.cls#OA\_versus\_normal\_repos |
| Upregulated in class | OA |
| GeneSet | KEGG\_GLYCOSAMINOGLYCAN\_DEGRADATION |
| Enrichment Score (ES) | 0.5174188 |
| Normalized Enrichment Score (NES) | 1.6442069 |
| Nominal p-value | 0.01010101 |
| FDR q-value | 0.031883117 |
| FWER p-Value | 0.407 |
Table: GSEA Results Summary

  

Fig 1: Enrichment plot: KEGG\_GLYCOSAMINOGLYCAN\_DEGRADATION      
 Profile of the Running ES Score & Positions of GeneSet Members on the Rank Ordered List

  

| SYMBOL | TITLE | RANK IN GENE LIST | RANK METRIC SCORE | RUNNING ES | CORE ENRICHMENT || 1 | SPAM1 | sperm adhesion molecule 1 [Source:HGNC Symbol;Acc:HGNC:11217] | 180 | 0.831 | 0.1237 | Yes |
| 2 | GLB1 | galactosidase beta 1 [Source:HGNC Symbol;Acc:HGNC:4298] | 337 | 0.731 | 0.2325 | Yes |
| 3 | GUSB | glucuronidase beta [Source:HGNC Symbol;Acc:HGNC:4696] | 904 | 0.541 | 0.2782 | Yes |
| 4 | HYAL1 | hyaluronidase 1 [Source:HGNC Symbol;Acc:HGNC:5320] | 997 | 0.518 | 0.3569 | Yes |
| 5 | HEXB | hexosaminidase subunit beta [Source:HGNC Symbol;Acc:HGNC:4879] | 1434 | 0.438 | 0.3955 | Yes |
| 6 | HPSE | heparanase [Source:HGNC Symbol;Acc:HGNC:5164] | 2360 | 0.319 | 0.3766 | Yes |
| 7 | HS3ST3A1 | heparan sulfate-glucosamine 3-sulfotransferase 3A1 [Source:HGNC Symbol;Acc:HGNC:5196] | 2637 | 0.286 | 0.4026 | Yes |
| 8 | NAGLU | N-acetyl-alpha-glucosaminidase [Source:HGNC Symbol;Acc:HGNC:7632] | 2767 | 0.273 | 0.4378 | Yes |
| 9 | ARSB | arylsulfatase B [Source:HGNC Symbol;Acc:HGNC:714] | 2787 | 0.271 | 0.4812 | Yes |
| 10 | HEXA | hexosaminidase subunit alpha [Source:HGNC Symbol;Acc:HGNC:4878] | 3025 | 0.247 | 0.5037 | Yes |
| 11 | HPSE2 | heparanase 2 (inactive) [Source:HGNC Symbol;Acc:HGNC:18374] | 3318 | 0.219 | 0.5174 | Yes |
| 12 | HS3ST3B1 | heparan sulfate-glucosamine 3-sulfotransferase 3B1 [Source:HGNC Symbol;Acc:HGNC:5198] | 4683 | 0.098 | 0.4277 | No |
| 13 | IDUA | alpha-L-iduronidase [Source:HGNC Symbol;Acc:HGNC:5391] | 5002 | 0.073 | 0.4151 | No |
| 14 | GALNS | galactosamine (N-acetyl)-6-sulfatase [Source:HGNC Symbol;Acc:HGNC:4122] | 5302 | 0.048 | 0.3998 | No |
| 15 | SGSH | N-sulfoglucosamine sulfohydrolase [Source:HGNC Symbol;Acc:HGNC:10818] | 5668 | 0.018 | 0.3744 | No |
| 16 | HGSNAT | heparan-alpha-glucosaminide N-acetyltransferase [Source:HGNC Symbol;Acc:HGNC:26527] | 5809 | 0.008 | 0.3648 | No |
| 17 | HYAL4 | hyaluronidase 4 [Source:HGNC Symbol;Acc:HGNC:5323] | 8209 | -0.172 | 0.2071 | No |
| 18 | GNS | glucosamine (N-acetyl)-6-sulfatase [Source:HGNC Symbol;Acc:HGNC:4422] | 9345 | -0.259 | 0.1619 | No |
| 19 | IDS | iduronate 2-sulfatase [Source:HGNC Symbol;Acc:HGNC:5389] | 10036 | -0.325 | 0.1621 | No |
| 20 | HYAL2 | hyaluronidase 2 [Source:HGNC Symbol;Acc:HGNC:5321] | 10420 | -0.363 | 0.1925 | No |
Table: GSEA details [plain text format]

  

Fig 2: KEGG\_GLYCOSAMINOGLYCAN\_DEGRADATION      
 Blue-Pink O' Gram in the Space of the Analyzed GeneSet

  

Fig 3: KEGG\_GLYCOSAMINOGLYCAN\_DEGRADATION: Random ES distribution      
 Gene set null distribution of ES for **KEGG\_GLYCOSAMINOGLYCAN\_DEGRADATION**
